# A new ground level neutron monitor for space weather assessment

**DOI:** 10.1038/s41598-024-57583-0

**Published:** 2024-03-26

**Authors:** Michael D. Aspinall, Tilly L. Alton, Cory L. Binnersley, Steven C. Bradnam, Stephen Croft, Malcolm J. Joyce, Dakalo Mashao, Lee W. Packer, Tony Turner, James A. Wild

**Affiliations:** 1 School of Engineering, Lancaster University, Lancaster, LA1 4YW UK; 2 Mirion Technologies (Canberra UK) Limited, 207A Cavendish Place, Birchwood Park, Warrington, WA3 6WU UK; 3 Culham Centre for Fusion Energy, United Kingdom Atomic Energy Authority (UKAEA), Abingdon, OX14 3DB UK; 4 Physics Department, Lancaster University, Lancaster, LA1 4YB UK

**Keywords:** Experimental nuclear physics, Solar physics

## Abstract

We report on a new ground-level neutron monitor design for studying cosmic rays and fluxes of solar energetic particles at the Earth’s surface. The first-of-its-kind instrument, named the NM-2023 after the year it was standardised and following convention, will be installed at a United Kingdom Meteorological Office observatory (expected completion mid 2024) and will reintroduce such monitoring in the UK for the first time since ca. 1984. Monte Carlo radiation transport code is used for the development and application of parameterised models to investigate alternative neutron detectors, their location and bulk material geometry in a realistic cosmic ray neutron field. Benchmarked against a model of the current and most widespread design standardised in 1964 (the NM-64), two main parameterisation studies are conducted; a simplified standard model and a concept slab parameterisation. We show that the NM-64 standard is well optimised for the intended large-diameter boron trifluoride (BF_3_) proportional counters but not for multiple smaller diameter counters. The new design (based on a novel slab arrangement) produces comparable counting efficiencies to an NM-64 with six BF_3_ counters and has the added advantage of being more compact, lower cost and avoids the use of highly toxic BF_3_.

## Introduction

We report on the progress made in modelling and measurements towards a new ground level neutron monitor (NM) design. This is a field where almost no progress in cosmic ray (CR) neutron detection techniques have been made since the 1960s.

CRs are high-energy (10^6^ eV to 10^21^ eV) subatomic particles, primarily protons ($\sim$90%), with some $\alpha$-particles and traces of heavier nuclei. They mostly originate from outside the solar system, likely stemming from explosive events such as supernovae, leading to the name galactic cosmic rays (GCRs)^1^. GCRs interact continuously with Earth’s atmosphere. Those with nucleon energies above about (300–400) MeV create secondary particles through atmospheric interactions, while lower-energy particles get absorbed in the upper atmosphere. High-energy secondary particles collide with air nuclei, generating a complex nuclear-electromagnetic-muon cascade known as an extensive air shower^2,3^.

Solar eruptive processes are another sporadic source of high energy particles. Two distinct physical mechanisms, magnetic reconnection acceleration during solar flares, and shock-wave acceleration as fast-moving coronal mass ejections (CMEs) move through interplanetary space, can accelerate ions to energies high enough to penetrate the terrestrial atmosphere^4^. The acceleration of particles in CME-related events typically lasts several days and exhibit larger fluences, while impulsive or flare-related events often have durations of a few hours and are associated with smaller fluences. Impulsive events are typically observed when the observer is magnetically connected to the flare site at the Sun, while ions accelerated at large-scale and expanding CME-driven shocks can populate magnetic field lines ahead of the CME over a wider region of space^5^. Termed solar energetic particles (SEPs), these can be accelerated to >1 GeV/nucleon and also produce a cascade of secondary particles. If such events reach ground level, they are termed ground level enhancements (GLEs). There are about ten GLEs observed per solar cycle^6^. However, if a CME engulfs the Earth, the relatively strong magnetic field within the ejected material can also shield terrestrial detectors from the CRs originating from outside the solar system, causing a reduction in the observed count rate known as a Forbush decrease^7^.

SEPs can degrade solar arrays, damage electronic components or cause single event effects and therefore can lead to significant disruption of critical infrastructure underpinning society and economy. Impacts may include disruptions to power, Global Navigation Satellite Systems (e.g., Global Positioning System (GPS)) and telecommunications (e.g., satellite communications and high-frequency radio), aviation (with an increase in background radiation doses at high altitudes and in space), and ground-based digital components^8–13^. Consequently, severe space weather features in the UK Government’s National Risk Register^14^. The UK Severe Space Weather Preparedness Strategy^15^ sets to build resilience to the risk of severe space weather by presenting the UK’s ambition, progress and policies to assess, prepare, respond and recover. The UK National Space Strategy^16^ identifies five goals and action that government, academia and industry will need to take to achieve them.

A GLE typically triggers a rapid surge in the flux of secondary fast neutrons across a broad expanse of the Earth’s surface , persisting for a duration of 15 minutes or more, before gradually subsiding back to the quiescent level. GLEs are studied using a global network of ground based neutron monitors (NMs). NMs were invented by Simpson (ca. 1948) to study primary CRs by detecting the secondary neutrons produced by CRs interacting in the Earth’s atmosphere^17,18^. The Climax NM started operating in 1951^19^, whereas many other NM stations were launched during the International Geophysical Year (IGY) in 1957/58 with the IGY NM designed by Simpson in the early 1950s^18^. Based on the collected experience, the design was improved, and a new type of detector, called the NM-64 or “super-monitor” (because of its increased counting rate), was introduced during the International Quiet Sun Year of 1964 and standardised by Carmichael^20,21^. Its stable operation and robust data production means it remains the standard design used today. The NM-64 is designed around the 14.5 cm diameter Chalk River BP28^22^ BF_3_ gas-filled proportional counters (however, a significant proportion of the global NM-64s adopt a Soviet equivalent of the BP28, the SNM-15, which has a $\sim$15% reduced efficiency^23,24^). They operate on the fundamental principles of the production of spallation neutrons in a target with high atomic weight (a lead (Pb) producer), the moderation of these fast neutrons (plus others from interactions in the body of the NM and its surroundings and some of the incoming fast neutrons) in a hydrogenous material (originally paraffin wax and now high-density polyethylene (HDPE)), and the detection of the thermal neutrons indirectly by ionising particles that are produced in a neutron induced nuclear reaction (typically BF_3_ gas filled proportional counters). A hydrogenous reflector material (again, initially paraffin wax but now HDPE) surrounds the monitor to reflect and moderate the evaporation neutrons that are produced in the Pb, and to shield and absorb the low energy neutrons that are produced by high energy nucleons in interactions with the materials surrounding the monitor^25^.

Approximately 50 operating stations, of mostly the 1964 Carmichael standard^20^, make up this network today^25,26^. Since the operation of the global network, the production of atmospheric secondaries, geomagnetic effects and NM response in general have been resolved. More recently, given the long-term reliability and automated data acquisition of the remaining NMs in the network, contributions towards spectral measurements, anisotropy studies and solar neutron measurements have been made^19^. In the present context, data from a network of NMs at various latitudes and longitudes are used by atmospheric radiation nowcasting models^27^ to retrieve information about the energy spectrum and the flux direction of the primary CRs for GLE alerting. Väisänen *et al.*^28^ provide a brief history of NM as space-physics instruments whilst providing a comprehensive analysis of all available NM data sets (300 data sets from 147 NMs spanning almost seven decades), considers the quality and consistency of the data and provides recommendations. Several other works document improvements made, standardisations and recommend further strategies to achieve the most optimal network^19,29–31^.

Since the widespread adoption of the NM-64, very little has changed until this work. One 2012 study maintains the standard IGY and NM-64 geometries and replaces the BP28-BF_3_ (due to concerns over the toxicity) with 2^″^ diameter ^3^He counters^32^; here we discover that these early standards are not optimised for smaller diameter detectors. Moreover, modern NM-64s again use BF_3_ because of the volatile price of ^3^He^25^, partly a result of overstated concerns over its supply^33^. Moreover, of the reported claim that ^3^He-filled counters appeared unstable over long terms because of high pressure and leaking ability of helium^28^, there is no evidence of ^3^He leaking from counters used in practice for safeguards, non-proliferation or reactor measurements. Besides these, only a few other alternative design approaches have been proposed^34,35^, which whilst cost effective and portable, their counting rates are not comparable to monitors in the established network. At present, BF_3_-filled proportional counters of slightly improved design (higher gas pressure) are used in the form of the mini-NM^31^. The Acute Exposure Guideline Level^36^ value for BF_3_ of lethal concentration (AEGL-3) is 110 mg  m^-3^ in 10 minutes. The BP-28 tube contains about 25 g of BF_3_, in a room 5 m $\times$ 10 m $\times$ 2.5 m (W $\times$ L $\times$ H) and uniformly dispersed this is 200 mg  m^-3^ , almost twice the lethal concentration. At the onset we wanted to achieve better neutron-economy and avoid BF_3_ to ease handling and future decommissioning, and to adhere to the modern regulatory environment, to simplify the safety case and satisfy political pressures.

UK Government awareness of the risks and drive to improve its capabilities for space weather monitoring and prediction provided the funding and motivation to reintroduce monitoring in the UK for the first time since the mid-1980s. The limited innovation and application of modern techniques offer significant opportunities for innovation in this field.

We introduce a new design (the NM-2023) based on a simple to fabricate slab arrangement that produces comparable counting efficiencies to an NM-64 in a more compact and lower cost design, and whilst avoiding the use of highly toxic BF_3_. The NM-2023 design operates on the same fundamental principles as the NM-64 as described (a fuller description is available at the beginning of the “Results” section). However, to our knowledge, for the first time since the widespread adoption of the NM-64, the bulk material has been re-imagined for optimal use with smaller diameter commercially available proportional counters. The new slab concept increases the packing density of neutron detectors and reduces neutron depletion in detector locations. Reducing the detector void and optimising the producer and moderator material for smaller diameter counters has eliminated the air-cavity around the circular Pb tube sections known as “rings” and simplified the design and manufacture, permitting the more compact, lower cost design.

We report on the development and application of parameterised radiation transport models to investigate alternative detectors, their location and bulk material geometry in a realistic CR neutron field to derive at an optimised design. We optimise on count rate response to a realistic field without consideration of the response to specific energies. Our simulations, computationally benchmarked against an NM-64 model, present relative comparisons between designs considering only the neutron component of the secondary cascade as the dominant event type. We considered this to be a reasonable and adequate approach as including the proton and muon component adds complication for little gain with a primary aim to explore alternative designs that match the 6-NM-64 neutron response with a reduced footprint, volume, mass and cost. In neglecting the proton and muon response, which is a minor contribution (and influenced heavily by site specifics), our predicted responses are assumed conservative given the additional neutron production as protons and muons interact with the Pb producer. In addition, any under prediction in response calculated for the NM-64 design will be closely equal to that of any under prediction in response calculated for our new design. Although environmental factors such as geomagnetic cutoff rigidity, elevation, atmospheric pressure and weather effects (e.g., snow) can affect count rates these are operational, site specific issues and do not dramatically influence the design of the instrument. However, the reflector material does provide some immunity to variations in the immediate environment, but weather effects are mitigated by the building that the instrument is housed in, e.g., lightweight with a steep pitched roof and heating, ventilation, and air conditioning^20^.

On that basis, here we present two main parameterisation studies; a parameterisation of a simplified NM-64 model, referred to as the cavity model, and a concept slab parameterisation. In a supporting study, we performed experiments using a standard ^3^He neutron counting module in reproducible, clutter-free geometries with radiation sources and in quiescent conditions over several weeks with the counting module encased in a Pb sarcophagus^37^. The latter provided an analogue to the design concept proposed here and data to validate our models and CR source term. We observed good agreement between experimental and simulated results and in count-rate trends recorded by NM-64 monitors in the existing network over the same period.

Sustained research motivation into ^3^He alternative neutron detector technology^38,39^ is a product of concerns over its supply, its volatile price and its extensive use in detectors for safeguards and security applications. One such alternative technology, that we identified as a potential for meeting the demanding application requirements of CR neutron monitoring, is based on boron-coated straws (BCS). This technology employs a low-cost method for coating the inner surface of long copper tubes, known as “straws”, with a thin layer of ^10^B-enriched boron carbide, then filled with an Ar/CO_2_ gas mix and operated as a proportional counter. In a supporting study, we established Monte Carlo N-Particle ^®^ (MCNP ^®^)^40^ models for two BCS detector configurations manufactured by Proportional Technologies, Inc. (PTI), Houston, Texas, and a commercial off-the-shelf (COTS) ^3^He detector (used in this study), and conduct experiments to validate our models and evaluate BCS-based detectors, benchmarking them against ^3^He detectors of an established type^41^.

The NM-2023 design is optimised for modern 1^″^^3^He counters and is benchmarked against a 6-NM-64 (the leading number denoting the number of BP28-BF_3_ counters, in this case a 6-counter NM-64). MCNP transport code simulations of the new design indicate an equivalent counting efficiency to a 6-NM-64 relative to the simulated CR neutron field. Moreover, this has been achieved with a 64% smaller footprint, 80% smaller volume and 55% of the mass, calculated based on dimensions and material volumes derived from the NM-2023 MCNP CAD models compared with the reported design parameters of the 6-NM-64^20^. It is estimated to be $\sim$50% cheaper than the present-day build costs of a 6-NM-64 based on predicted fabrication cost reductions, achieved by a simpler slab design rather than several Pb and polyethylene rings , and reduced cost of raw materials (Pb and HDPE) given the reduced material volume. The quoted cost-saving estimate is considered cautious, as it relates only to the instrument and does not account for cost savings associated with to housing, infrastructure and deployment of a smaller instrument.

## Results

**Figure 1:**
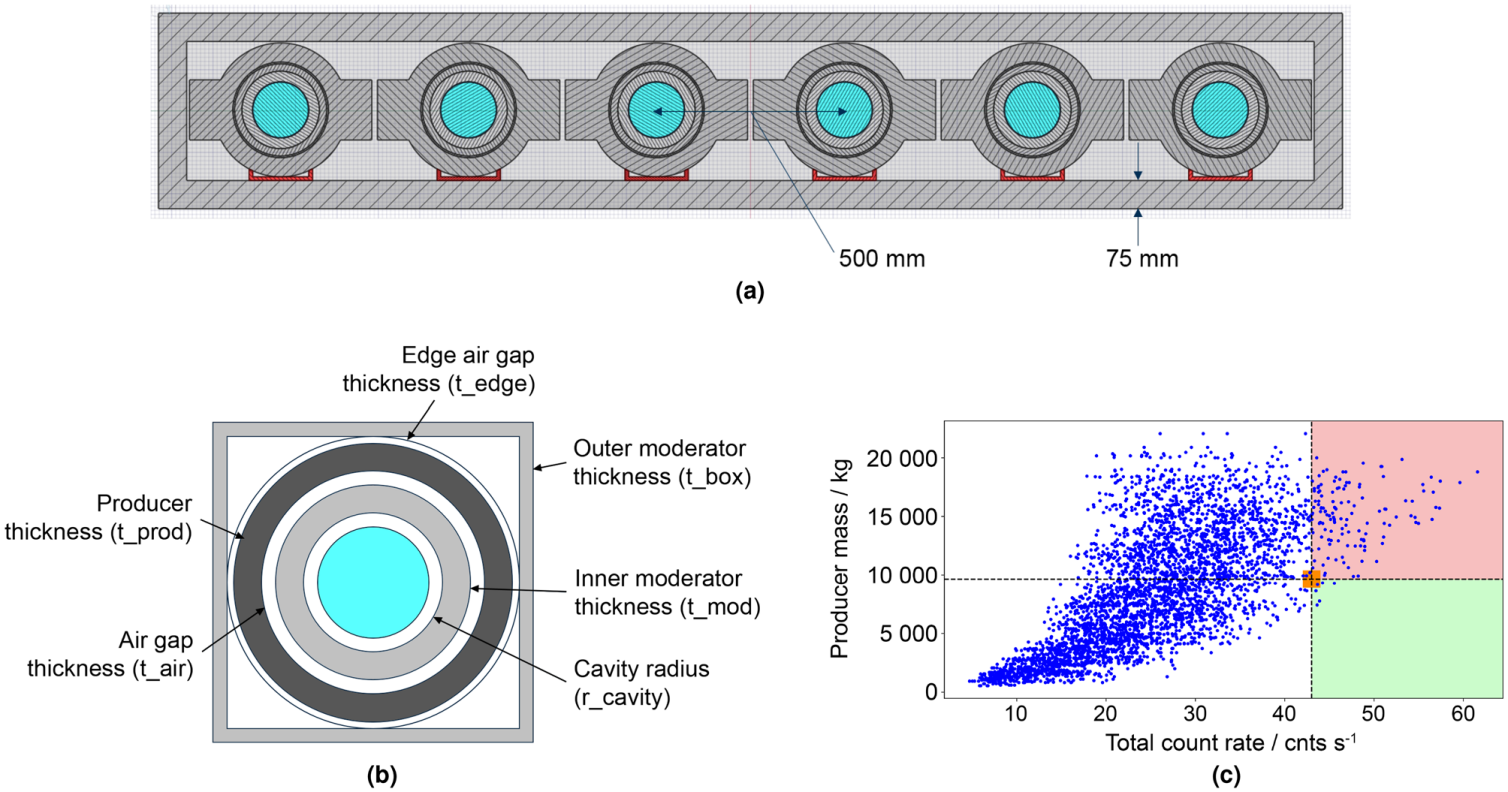
6-NM-64 parameterisation. (**a**) Vertical cross section (to scale) of the radiation transport model developed and used to represent the 6-NM-64 benchmark. Moving from the inside out, comprising six 6^″^ dia. BP28-BF_3_ proportional counters (blue), air gap, 2 cm thick HDPE moderator tube and 5.1 cm thick *winged* Pb rings (one per counter) and outer 7.5 cm thick HDPE reflector with dimensions as indicated. (**b**) Single counter representation of the parameterised cavity model used as an equivalent to the NM-64 standard with key parameters indicated. NB: this figure represents a single cavity; all simulation performed in this study were for six cavities as per the 6-NM-64 benchmark. The wings of the Pb rings were omitted from the standard NM-64 design for simplicity. (**c**) Producer mass in kg against total (i.e., summed across all six detectors) count rate in cnts s ^-1^ for randomly generated parameter configurations of the parameterised cavity model shown in (**b**). The orange marker, located at the intercept of the orthogonal dashed lines, indicates the 6-NM-64 benchmark performance; a count rate of $\sim$43 cnts  s^-1^ for a producer mass of $\sim$10 tonne. The region shaded red signifies parameter configurations that exceed the count rate performance of the 6-NM-64 benchmark but for a greater producer mass. The region shaded green signifies parameter configurations that exceed the count rate performance of the 6-NM-64 benchmark but for a lower producer mass. The few parameter configurations in the region shaded green indicates a well optimised NM-64 configuration.

The *cavity* model is a descriptive name we gave to the NM-64 design as it relies on cavities created by Pb rings in which moderated thermal neutron detectors (typically BP-28 BF_3_ counters) are located (Fig. 1a). Incoming CR nucleons, for the most part, pass through the outer polyethylene reflector material unperturbed. The purpose of the outer reflector is to shield and absorb the low energy neutrons that are produced by high-energy nucleonic interactions with the materials surrounding the monitor. A CR nucleon that passes through the reflector then may undergo a violent nuclear reaction (known as spallation) with the heavy Pb nucleus of the producer material. In the context of the present discussion, spallation occurs when a relativistic light, but very high-energy particle (a proton or a neutron) hits a heavy nucleus causing it to disintegrate through inelastic nuclear reactions. The disintegration of the Pb nuclei results in the emission of neutrons and other particles, essentially amplifying the response to be measured. The reflector material has a dual function, as well as isolating from environmental influences, it moderates and reflects the neutrons produced by the reactions in the Pb producer inwards towards the neutron detectors. Neutrons generated during the spallation reaction are then thermalised by the polyethylene moderator surrounding the thermal neutron detectors. The thermal neutrons are then detected indirectly by ionising particles that are produced in a neutron induced nuclear reaction (for detectors employing BF_3_, thermal neutrons captured in the ^10^B are converted into secondary particles through the ^10^B(n,$\alpha$)^7^Li reaction, while detectors employing ^3^He, thermal neutrons are converted into secondaries through the ^3^He(n,p)^3^H reaction).

The *slab* model, presented here for the first time, describes a configuration of reflector, producer and moderator arrangement that resembles a slab (Fig. 5), i.e., it eliminates the producer and moderator rings employed by the cavity model. Instead, a cuboidal polyethylene moderator slab with uniformly distributed detectors (received via machined bores in the moderator) is encased in a cuboidal Pb sarcophagus before being encased in an outer layer of polyethylene reflector. This affords greater flexibility, simplifies fabrication (so reduces cost) and increases the sensor packing density by eliminating the voids inherent in the cavity model. The slab concept still relies on the fundamental detection physics of the NM-64 (cavity) design.

### NM-64 benchmark

To establish a modelling benchmark, a 6-NM-64 radiation transport model was developed based on the reported design parameters^20^. A CAD model was first devised in Ansys ^®^ SpaceClaim^42^, with material types classified by colour. The model conformed to constructive solid geometry without any modifications and was subsequently converted into MCNP geometry. The integrity of the translated geometry was tested for lost particles using a void run and zero lost particles were identified in 10^9^ source particles, indicating a well defined geometry for radiation transport calculation. Figure 1a shows a vertical cross section of the 6-NM-64 benchmark model. A benchmark count rate was derived using the 6-NM-64 model and source term (detailed in the “Methods”), running for $10^7$ source particle histories with results presented in Table 1.

### NM-64 equivalent comparisons

To create a 6-NM-64 equivalent using the parameterised cavity model (Fig. 1b and described in the “Methods” section), the 6-NM-64 dimensions^20^ were used to determine values for the cavity radius ($r\_cavity$), the inner moderator thickness ($t\_mod$), the air gap thickness ($t\_air$) and the outer moderator thickness ($t\_box$). In the cavity model, the *wings* of the Pb rings were omitted from the standard NM-64 design, a producer thickness ($t\_prod$) value was calculated such that an equivalent producer mass is conserved between the 6-NM-64 benchmark and equivalent cavity model. The producer mass in the benchmark 6-NM-64 is 9650 kg^20^, corresponding to a $t\_prod$ value of 6.77 cm in the equivalent model.

The equivalent model was ran for 10^7^ source particles and compared to the benchmark model. The output data are compared in Table 1, the statistical errors on the total count rate for both models overlap, indicating that removing the wings from the NM-64 benchmark design is statistically negligible. To simplify the engineering of any potential cavity based design, the wings could therefore be removed from the design. The wings were originally included to provide a uniform Pb thickness for use with a meson monitor placed below it, making use of the Pb as a soft component absorber (our slab design achieves this naturally)^20^.

**Figure 2:**
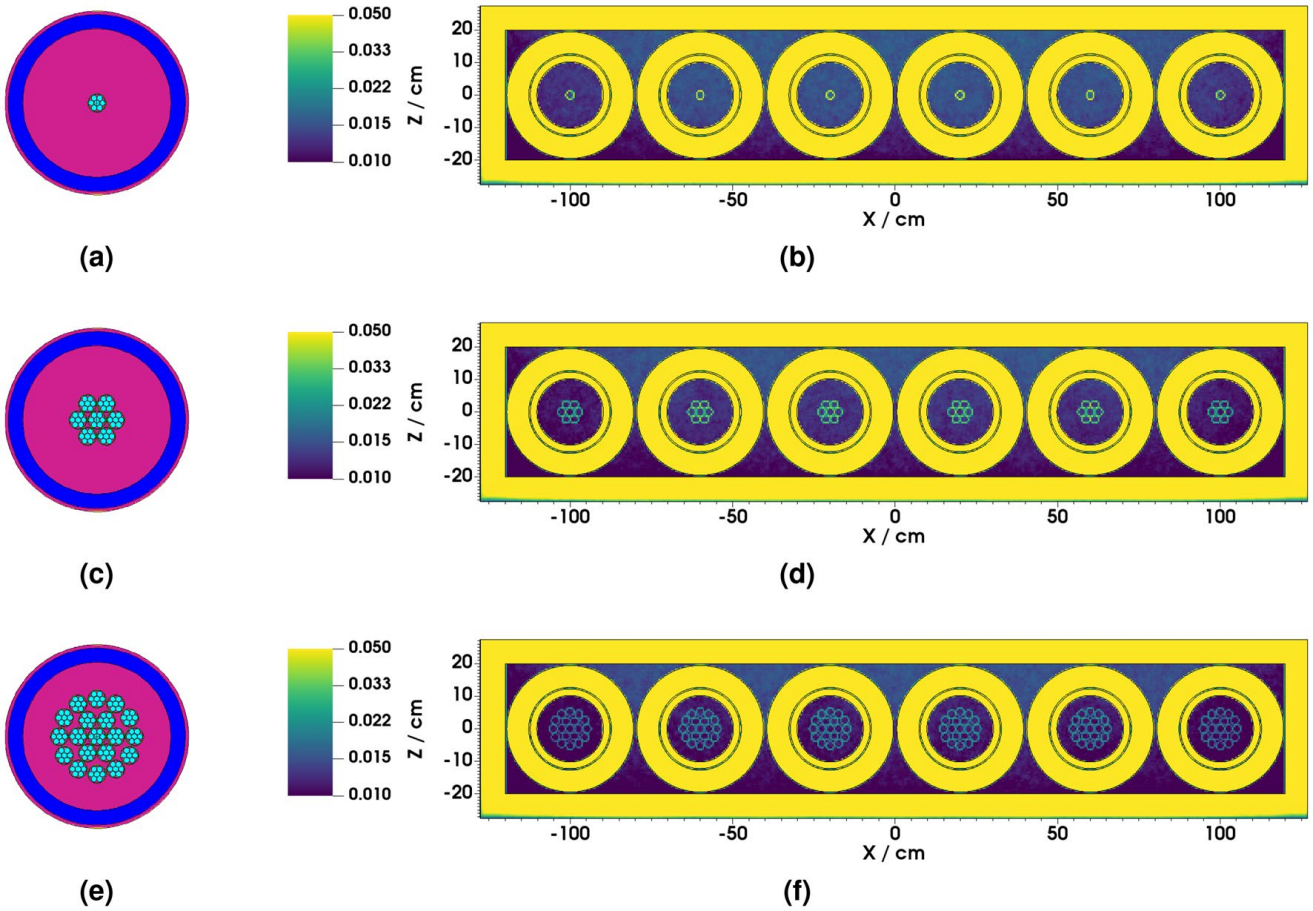
NM-64 equivalent parameterised cavity model filled with (**a**) 1, (**c**) 7 and (**e**) 19 PTI-204 BCS detectors per cavity. Indicative spatial distribution of the ^10^B(n,$\alpha$) reaction rate tally with (**b**) 1, (**d**) 7 and (**f**) 19 PTI-204 BCS detectors per cavity. Intense yellow indicating a high relative reaction rate, dark blue indicating a low (depleted) reaction rate.

In considering BCS-based detectors as an alternative to the BF_3_ counters used in the original NM-64, multiple 1^″^ dia. PTI-204 BCS detectors^41^ were modelled in the cavity of the 6-NM-64 equivalent model. The PTI-204 detector is made up of seven round 7.5 mm diameter boron coated straws encased in a 1^″^ dia. aluminium tube. Each straw element in the PTI-204 detector represents mini proportional counters. Straws are tied together to give one signal output and supplied with an integrated amplifier and high voltage supply^43,44^. The BCS tubes were extended to the same length as the BF_3_ counters to provide a direct comparison. Simulations were ran for 1, 7 and 19 tubes per cavity to count surplus thermal neutron production which may exist within the cavity. The 1, 7 and 19 tube configurations are shown in Fig. 2a,c,e, respectively.

Table 1 compares the relative count rate for the NM-64 equivalent cavity model with 1, 7 and 19 PTI-204 BCS tubes per cavity against the 6-NM-64 benchmark data, each simulation was ran for $10^7$ source particles. The relative count rate increase drops off as a function of the number of tubes per cavity, for example, the count rate for 7 tubes per cavity is less than 7 times higher than 1 tube per cavity. The count rate achieved with 19 tubes per cavity exceeds the benchmark 6-NM-64 count rate by a statistically significant margin. However, when considering the spatial distribution of reaction rate per unit volume (Fig. 2b,d,f), the 19 tube configuration (Fig. 2e) does not appear to be the optimum use of those detectors; intense yellow indicating a high relative reaction rate, dark blue indicating a low (depleted) reaction rate. Here a neutron mesh tally was calculated over the full detector, with a ^10^B(n,$\alpha$) reaction rate tally multiplier applied to the mesh. The results therefore provide an indicative spatial distribution of neutron detection probability, which is directly proportional to the ^10^B(n,$\alpha$) reaction rate. Figure 2b,d,f shows that a potentially higher reaction rate in some of the tubes could be achieved if they were moved elsewhere in the assembly, thus the 19 tube per cavity configuration is sub-optimal.

### Randomised parameter scan

To explore the full parameter space of the parameterised cavity model, parameters were randomly generated within specified ranges (shown in Table 2) to construct 1000 unique models for the BP28-BF_3_ counter. Fig. 1c shows the resulting scatter plot of the producer mass against the total count rate. The 6-NM-64 baseline count rate (43.04 cnts  s^-1^), indicated by the orange marker, is exceeded by many of the configurations derived. However, only 7 of these have a lower Pb mass than the 6-NM-64 standard, which indicates a well optimised NM-64 configuration and prompted our investigations into alternative slab configurations.

**Table 1:** Comparison of summed count rate in cnts s^-1^ across all six cavities for the 6-NM-64 benchmark model (Fig. 1a), the 6-NM-64 equivalent cavity model (Fig. 1b shows an example single cavity) and the cavity model with the BP28-BF_3_ detectors replaced with 1, 7 and 19 PTI-204 BCS detector(s) per cavity (Fig. 2a,c,e shows the 1, 7 and 19 detector configurations, respectively).

6-NM-64 BP28-BF_3_			PTI-204 BCS		
Benchmark	Equivalent	$\Delta$	1 detector	7 detectors	19 detectors
$43.04 \pm 0.50$	$43.86 \pm 0.51$	$1.89\%$	$8.58 \pm 0.08$	$31.13 \pm 0.15$	$47.17 \pm 0.18$

**Table 2:** Parameter ranges for the randomised parameter scan of the NM-64 equivalent parameterised cavity model depicted in Fig. 1b, generating 1000 unique cavity models utilising the BP28-BF_3_ counter.

Parameter	Range
$r\_cavity\_bounds$/cm	7.6–14.0
$t\_mod\_bounds$/cm	0.5–6.0
$t\_air\_bounds$/cm	0.1–3.0
$t\_prod\_bounds$/cm	0.5–10.0
$t\_edge\_bounds$/cm	0.1–3.0
$t\_box\_bounds$/cm	2.0–20.0

The results are shown in Fig. 1c.

**Table 3:** Fixed and varied (bold typeface) parameter values for the parameterised slab model for the (A) constant slab volume simulation, (B) equal lead thickness simulation and (C) detector location optimisation simulation.

Parameter	A	B	C
*width*/cm	<**400**	400	400
*height*/cm	<**200**	20	20
$t\_base$/cm	5	**0.5–20.0**	7
$t\_side$/cm	5	**0.5–20.0**	9
$t\_prod$/cm	15	**0.5–20.0**	10
$t\_gap$/cm	1	1	**0.25–1.50**
$t\_box$/cm	7.5	7.5	7.5

### Slab parameter optimisation

To determine the potential neutron detection efficiency of a given slab configuration (shown in Fig. 8a), a cell tally was added to the moderator block, with a ^10^B(n,$\alpha$) reaction rate tally multiplier added. Whilst this is not a direct measure of neutron detection efficiency, the global reaction rate calculated within the moderator provides a basic figure of merit (*FOM*) and relates to the potential efficiency. The *FOM* is defined by Eq. (1). Using this, three separate parameter studies were carried out to optimise the slab parameters; constant volume, equal Pb thickness and constant Pb mass. For all of these simulations, a 1 m active length detector was assumed.

1 $$\begin{aligned} FOM = N_{B_4C} \cdot V_{B_4C} \int ^\infty _0 \sigma _{B_4C}(E) \cdot \phi (E) {\cdot dE } \end{aligned}$$

where $N_{B_4C}$ is the number density of boron carbide inside the BCS (assuming 96% ^10^B enrichment). $V_{B_4C}$ is the volume of boron carbide inside each BCS. $\sigma _{B_4C}(E)$ is the total absorption cross section for the boron carbide ($\gg$99% of this is the ^10^B(n,$\alpha$) reaction). $\phi (E)$ is the average flux over the full inner moderator volume. The integral is directly computed in MCNP and the multiplier outside the integral is the quantity we define as the tally normalisation factor. The *FOM* is directly related to the count rate we would measure on an individual BCS, however it is based on the average flux over the entire inner moderator slab. Local variations in reaction rate are determined later using the mesh-based approach.

**Figure 3:**
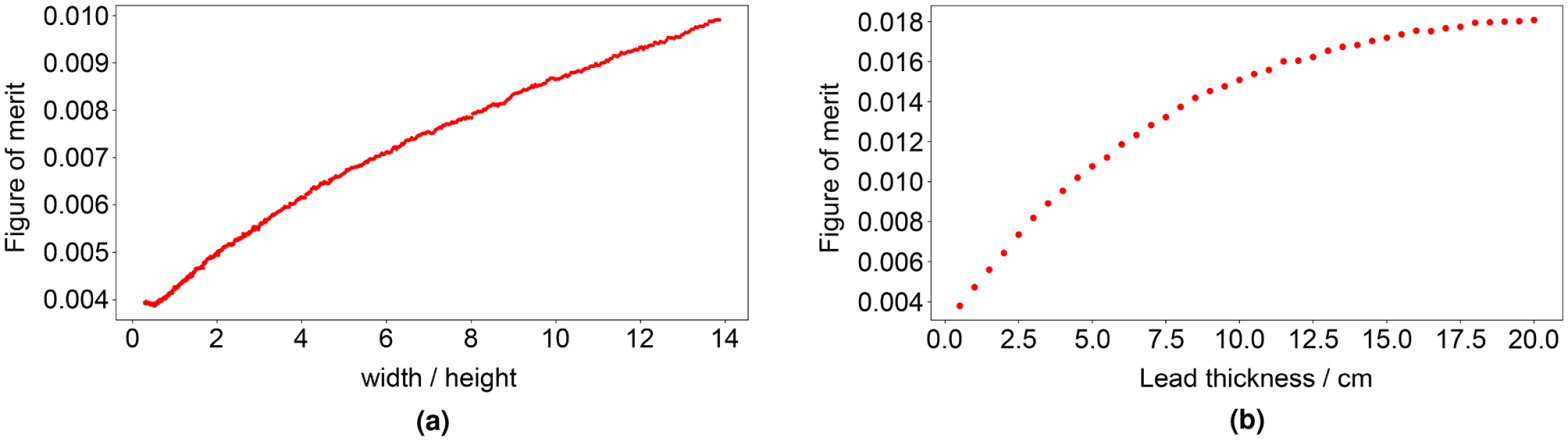
Results of the initial slab parameter optimisations showing the figure of merit (FOM, i.e., the indicative count rate from the ^10^B(n,$\alpha$) reaction rate tally) against (**a**) the aspect ratio for all constant volume simulations and (**b**) the Pb thickness in cm for all equal Pb thickness simulations.

Table 3, column (A), presents the fixed and varied parameters for the constant slab volume simulations, the cross sectional area of the moderator is constrained to equal the internal cross sectional area of the baseline 6-NM-64 (296 cm $\times$ 39 cm). 500 simulations were performed, with an upper limit of 4 m for the width and 2 m for the height, the aspect ratio was varied between these limits. Figure 3a shows a plot of a FOM (i.e., indicative of the count rate from the ^10^B(n,$\alpha$) reaction rate tally) against the aspect ratio.

**Figure 4:**
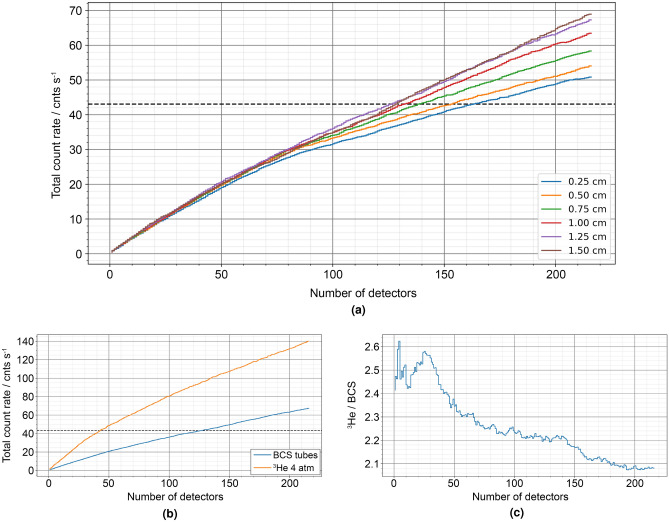
Results of the detector location optimisation simulations within the parameterised slab model showing calculated total count rate in cnts s^−1^ against (**a**) number of PTI-204 BCS detectors added for all $t\_gap$ simulations performed and (**b**) number of 4 atm ^3^He and PTI-204 BCS detectors for $t\_gap$ = 1.25 cm. The dashed horizontal line shows the 6-NM-64 benchmark count rate for comparison. (**c**) Shows the ratio of ^3^He / BCS total count rate against number of detectors for $t\_gap$ = 1.25 cm.

The largest aspect ratio yields the greatest count rate. On this basis, fixed parameter values for the equal Pb thickness simulations are given in Table 3, column (B). Here the parameters corresponding to the Pb box geometry shown in Fig. 8a, i.e., the topside ($t\_prod$), the side ($t\_sides$) and the underside ($t\_base$) thickness of the Pb producer, are set equal each other and are then varied from 0.5 to 20.0 cm in intervals of 0.5 cm. Figure 3b shows a plot of the FOM against Pb thickness.

As observed in Fig. 1c, the count rate increases as Pb mass increases. However, the relationship is non-linear as illustrated by Fig. 3b, the rate of count rate increase slows significantly beyond $\sim$10 cm.

For the next parameterisation study, a Pb mass of 10000 kg was used, a round figure derived from the perceived optimal 10 cm thickness. Fixed parameter values are given in Table 3, column (C). Keeping the Pb mass constant, $t\_prod$, $t\_side$ and $t\_base$ were varied to determine the optimal ratios to best utilise the given Pb mass. $t\_base$ was incremented on an inner loop of length 22, from 0.5 to 9.93 cm, where 9.93 cm has been calculated from $t\_base$ = $t\_side$ = $t\_prod$ = 10000 kg of Pb. $t\_side$ was incremented on an outer loop of length 22, from 0.5 to 9.93 cm, a total of 484 simulations (22 $\times$ 22).

Further parameterisations derived an optimal ratio of 0.7 ($t\_base$) : 0.9 ($t\_side$): 1 ($t\_prod$). However, the absolute producer mass has more impact on the FOM than its spatial distribution around the inner moderator block.

#### Detector location optimisation

Using the optimal slab parameters derived, the original cell tally used on the inner moderator block was replaced with a 5 cm resolution mesh tally in width and height, with the same ^10^B(n,$\alpha$) reaction rate tally multiplier added. This modification allows for local FOM determination within the moderator slab, which can be utilised to inform the optimal location within the moderator to position a neutron detector. An automated process was developed to populate the moderator with neutron detectors in optimal locations. The population methodology is illustrated in Fig. 8b,c. An integrated model generation and output data interpretation process was developed, utilising a mesh tally file reader to process the output data from each iteration. This allowed the maximum voxels not already occupied with a neutron detector to be identified and a detector to be placed in that location in the next iteration.

Iterations were performed for up to 216 neutron detectors on a 400 cm $\times$ 20 cm $\times$ 100 cm moderator block. Fixed parameters for all simulations were derived from the analysis described thus far and are defined in Table 3, column C, with repeated iterations carried out for an air gap thickness ($t\_gap$) between the outer reflector and the producer, and the producer and the inner moderator (see Fig. 8a) of 0.25 to 1.50 cm in 0.25 cm intervals, a total of 216 $\times$ 6 simulations.

The count rate distribution across the detectors was found to be more even for the 1.5 cm gap than for the 0.25 cm gap case. This effect is likely due to over moderation in the 0.25 cm gap case; neutrons which enter the detector from above undergo significant moderation, i.e., the mean free path is much shorter than the height of the moderator, and are subsequently mainly absorbed by the top row of detectors. In the 1.5 cm gap case, neutron absorption by the moderator appeared much lower, therefore the overall efficiency of the individual detectors appears to be greater, given that only 129 detectors were required to match the 6-NM-64 performance compared to 164 detectors for the 0.25 cm gap case. In both cases, the preferential location for the addition of neutron detectors appears to be the top row, which implies the optimal geometry would be thinner than that used for these simulations.

From Fig. 4a, the impact of increasing the gap size is shown for an increasing number of detectors; beyond 50 detectors, the increase in count rate observed for smaller gap sizes appears to be less than observed for larger gap sizes. For a similar count rate to the 6-NM-64 benchmark (marked with a black dotted line), 1.25 cm appears the optimum gap size; with this gap size, a count rate of 10 cnts  s^-1^ more is observed for the same number of detectors added with a gap size of 0.25 cm.

Assuming 1.25 cm as the optimum gap size and retaining the fixed parameters defined in Table 3, column C, a comparative study was performed between the 1^″^ dia. PTI-204 BCS and an equivalent ^3^He detector at 4 atm fill pressure. Figure 4b shows a plot of the total count rate against number of detectors for the PTI-204 BCS and the ^3^He detectors. Figure 4c shows a plot of the ratio of ^3^He to BCS count rates against number of detectors.

Figure 4b shows an initial steeper count rate increase for the ^3^He detectors in up to around 40 detectors, with a slower rate of increase beyond this. By comparison, the BCS count rate increase appears more linear. The key result is the ratio between the ^3^He and BCS count rates illustrated in Fig. 4c, which initially peaks to around 2.6 during the initial ^3^He count rate increase described and remains above 2 for all detectors added. Comparing the efficiency per detector added (Fig. 3b), the factor of increase from BCS to ^3^He approaches 3 for 150 BCS tubes against 50 ^3^He tubes (rounded-up tube numbers which achieve a count rate greater than the 6-NM-64 benchmark).

Despite the higher cost of ^3^He, the potential design benefits of higher efficiency outweighs the additional cost per detector when considering all cost implications (e.g., additional Pb, polyethylene and electronics to accommodate a greater number of detectors) and long term (>50 years) operating potential. Building on this, detector characteristics that influence relative efficiency (and cost) were considered in achieving optimum efficiency/cost. BCS efficiency inherently depends on the B_4_C deposit thickness, whereas ^3^He efficiency is dictated by gas fill pressure. The details of this study are not included here, but based on a detailed engineering breakdown and trade-off arguments, ^3^He at 4 atm fill pressure is most cost-effective^41^.

#### ^3^He detector optimisation

**Figure 5:**
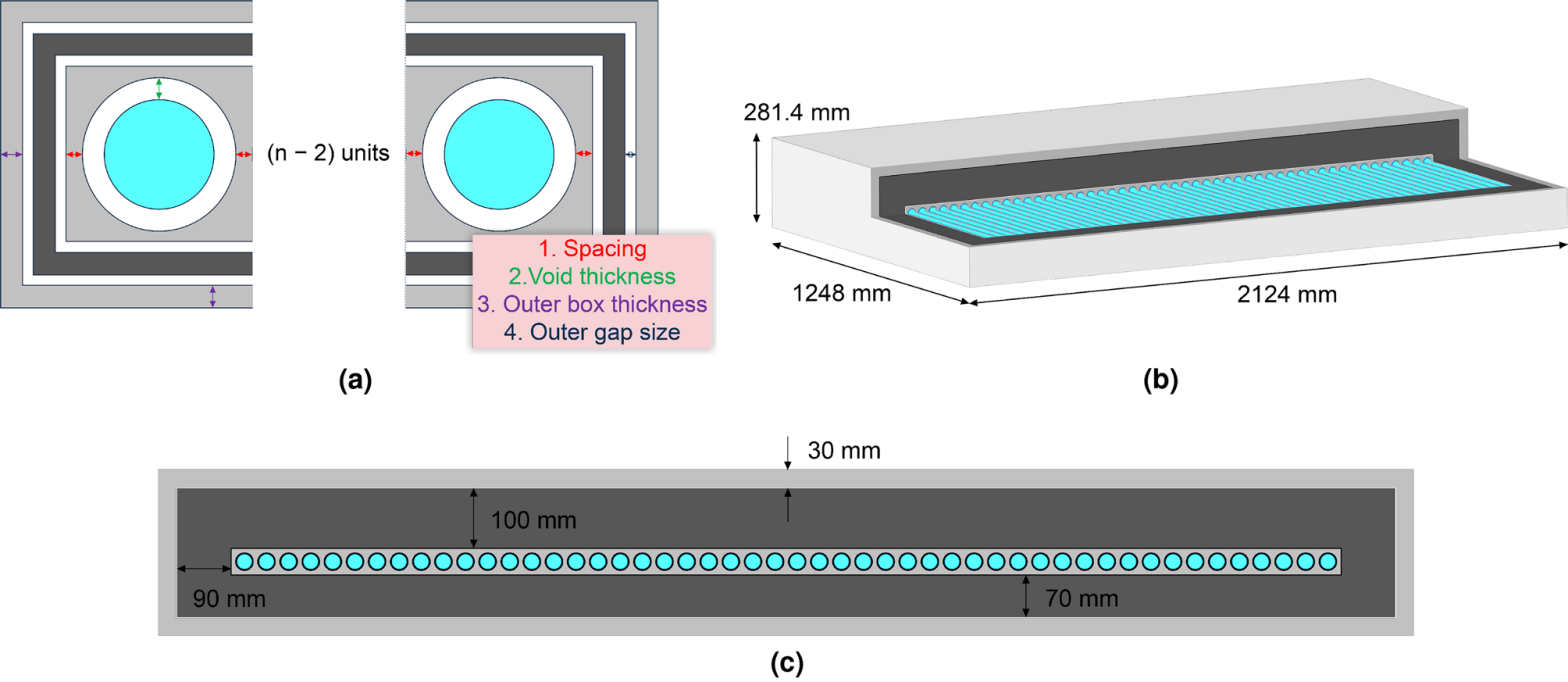
(**a**) Schematic of the parameterised slab model used for the ^3^He detector optimisations. 50 tubes were used for these simulations, the additional 48 tubes present in the centre of the model have been omitted for clarity. Parameters optimised included: 1. the spacing between detectors, 2. the air void thickness around the detector, 3. the outer reflector box thickness and 4. the air gap between the reflector and Pb producer. Parameters were optimised in order of predicted impact each would have on the overall detection efficiency. (**b**) Cutaway isometric (to scale) of the ^3^He optimised model with key dimensions as indicated. (**c**) Vertical cross section (to scale) of ^3^He optimised model with key dimensions as indicated.

Given the higher detection efficiency of ^3^He versus BCS, and therefore an overall smaller instrument size, further parameter optimisations were conducted to maximise the count rate per unit mass of producer for a fixed number of detectors. 50 1 m-long ^3^He detectors at 4 atm were chosen (from Fig. 4b, 44 ^3^He detectors equals the 6-NM-64 benchmark count rate). Figure 5a presents a visualisation of these parameter optimisations, which included the spacing between detectors, the air void thickness around the detector, the outer reflector box thickness and the air gap between the reflector and Pb producer. Parameters were optimised in order of predicted impact each would have on the overall detection efficiency (i.e., the most significant was optimised first and so on), the principle being that the less significant parameters would act only as *fine tuning* of the model. The Pb producer thicknesses were retained from the previous BCS optimisations, using $t\_base$ = 7 cm, $t\_side$ = 9 cm and $t\_prod$ = 10 cm. Iteration results are presented in Table 4, with a CAD representation of the optimised model presented in Fig. 5b,c.

**Table 4:** ^3^He detector optimisation input parameters and output dimensions and specific count rates.

	Iteration 1:	Iteration 2:	Iteration 3:	Iteration 4:
	Spacing	Void thickness	Outer box thickness	Outer gap size
*Spacing*/cm	**0.6**	0.6	0.6	0.6
*Void* *thickness*/cm	1.25	**0.3**	0.3	0.3
*Outer* *box* *thickness* /cm	7.5	7.5	**3**	3
*Outer* *gap* *size*/cm	1	1	1	**0.2**
*Moderator* *width*/cm	282.6	190.15	187.6	187.6
*Moderator* *height*/cm	6.24	4.44	4.34	4.34
*Specific* *counts*/cnts s^-1^ kg^-1^	0.00744	0.00841	0.008760	0.00916

Parameter values derived from a given iteration are indicated in bold (before the engineered concessions, detailed the Engineering optimisations section, have been applied).

#### Engineering optimisations

**Figure 6:**
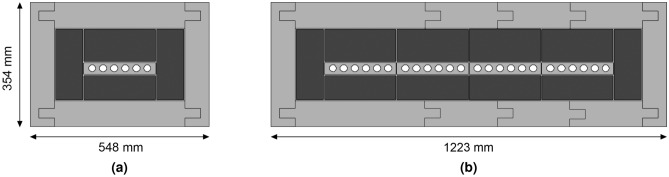
Modular engineered design solution with concessions applied, showing two of the possible four configurations with key dimensions indicated in mm. Following the nomenclature x-NM-2023, where x is the number of banks of six 1^″^ dia. ^3^He detectors and 2023 being the year designed, (**a**) the quarter-size monitor comprising of 1 bank of six detectors (1-NM-2023) and (**b**) the full-size monitor comprising of 4 banks of six detectors (4-NM-2023).

The optimised model was evaluated and analysed by mechanical engineers to derive at a final design that is practical, cost effective and that can be manufactured easily. Figure 6 presents the engineered design solution.

The most significant design concession was the use of 2 m long (nominal) ^3^He detectors (Reuter-Stokes RS-P4-0878-201)^45^ rather than the 1 m long detectors used during the optimisations. However, the producer mass (which is shown here to have the most influence over count rate performance) was conserved, essentially halving the width (and number of detectors) and doubling the depth (and length of detectors) with anticipated minimal impact from a neutronics perspective. In engineering terms, this concession halved the number of readout channels, reducing cost and complexity. To fulfil a requirement to modularise the design, 25 detectors were reduced to 24. This allowed detectors to be grouped into four banks of six detectors, providing an even number of detectors per bank and allowing detectors to be paired to a single preamplifier, again reducing cost and complexity. Figure 6a shows a quarter sized monitor and Fig. 6b shows a full size monitor design to equal the count rate of a 6-NM-64 and how the modularisation is achieved in quarter increments. All bulk material components were also modularised for ease of handling and installation, each with a mass <35 kg. The few millimetre gaps between the Pb producer and moderator, and reflector and producer were eliminated to reduce engineering complexities and cost. The analysis presented indicates that this will have minimal impact on performance. The outer reflector thickness was also increased to 7 cm following further analysis, making it equal to the thickness used by the NM-64.

## Discussion

The challenge was to re-establish CR NM capability in the UK taking advantage of modern design tools and improvements in nuclear instrumentation. The new NM (named the NM-2023) will be integrated as an operational instrument into the global network and this research is an essential part of communicating the design process to the international community. It is hoped future examples of the NM-2023 design will be built and used in other places both in the UK and in other countries. During which, the monitor’s response function with latitude will be established to allow the expanded array to work as a distributed spectrometer.

A condition of our research was to deliver an operational instrument. Considering this, we have adopted COTS components were possible, but have also investigated novel detection methods with some service history and evaluated the most promising candidates. Furthermore, the designed solution would need to meet the challenging application requirements of CR neutron monitoring (i.e., long term reliability and stability, system longevity and mitigable environmental influence) and be commercially viable. Considering these and given the similar application requirements for nuclear safeguards techniques and equipment , we employed similar modelling approaches for optimisation and detection technologies. In this regard, COTS components are attractive when we can identify a long-term supply chain, where other demanding users have helped establish reliability and non-recoverable engineering costs can be avoided. Our solution capitalises on the established nuclear safeguards supply chain and International Atomic Energy Agency (IAEA) approved-for-use technology were possible^46^.

A range of modelling activities have been presented which sought to identify key performance factors and guide a new NM design that is more compact, cheaper and capable of producing comparable results to the current NM-64 standard designed around legacy BF_3_ proportional counters. BCS and ^3^He detector options were evaluated as an alternative to BF_3_ counters as part of this exercise. We show that the NM-64 standard is optimised for the intended large-diameter BF_3_ counters but not for multiple smaller diameter detectors. The NM-2023 design, based on a slab arrangement, produces comparable counting efficiencies to a 6-NM-64 and is more compact (64% smaller footprint and 55% of the mass). Based on reduced fabrication and raw material costs it is estimated to be $\sim$50% cheaper than the present-day build costs of a 6-NM-64 and avoids the use of toxic BF_3_.

A computational NM-64 benchmark model was derived to evaluate relative performance of design concepts as they evolved. To do this, a methodology was developed to create a realistic CR neutron source term from the MAIRE database for use in MCNP. This provided flexibility over input data, but with limited resolution of directional distribution. Still, it was preferred over the MCNP integrated CR neutron source as this only contains basic directional dependency on the incident neutron energy. In evaluating the final engineered solution, once all source parameters are fixed, it would be advantageous to adopt a high resolution source term derived by transporting CRs directly to allow the spatial and directional distributions of secondary neutrons to be recorded. A BF_3_ version of the 6-NM-64 geometry was developed in CAD from original drawings and converted to a radiation transport model. Evaluated under our CR neutron source term, it provided a benchmark count rate of 43.04 cnts s^-1^.

A parameterised cavity model was developed and investigated. Based on the NM-64 design but simplified by removing the Pb wings, we show that the wings have minimal impact on the neutron count rate. A parameter scan was conducted using randomly generated parameters within fixed bounds (Table 2). Whilst the benchmark NM-64 count rate was improved by several configurations, only a few achieved this with marginally less Pb mass. This suggests that the Pb economy of the baseline NM-64 configuration, used with the intended large-volume BP28-BF_3_ detectors, is very well optimised.

An alternative slab configuration was explored. After deriving a set of parameters to constrain the slab geometry, the optimisation process was split into two workflows. The first involved the optimisation of the slab geometry, such as its dimensions, and the relative thicknesses of the producer, moderator and other features. Once an optimal-dimension slab was defined, a second workflow was implemented to assess the best location to position individual BCS detectors within the slab to maximise the count rate. We determined that the solution yielding the highest count rate has a large aspect ratio, with detectors equally spaced in the moderator in a single row. It was found that the Pb producer should ideally be distributed around the moderator block; its mass being the most significant parameter. However, small gains in efficiency were obtained by slightly adjusting the top layer thickness such that it is slightly thicker that the side and bottom thicknesses.

Using the optimal slab configuration, a comparison was performed between the PTI-204 BCS and 1^″^ external dia., 304 stainless steel body, 4 atm (25 ^∘^C) ^3^He detectors^45^. We determined that a significant gain in efficiency could be achieved using ^3^He detectors of a viable design concept, despite the higher cost per detector unit. The count rate per unit mass of Pb for ^3^He detectors was optimised, arriving at a compact concept model with a simulated count rate of 45.8 cnts s^-1^ ; exceeding the 6-NM-64 baseline whilst reducing the Pb mass by $\sim$50%, the monitor width by $\sim$30%, and height and depth by $\sim$50%.

To validate the simulations presented and experimentally evaluate the BCS detectors, several experiments were performed in parallel with the modelling activities^37,41^. Whilst these provide confidence in the modelling data for the neutron energies available during the experiments, validation of results at higher energies, where MCNP relies on models rather than nuclear data, is currently limited. This could be achieved by performing further experiments to measure long background neutron count rates in multiple locations for varying producer and moderator thicknesses.

Detailed final design analysis of the engineered solution using our validated models will now be undertaken. This would benefit from further testing using a higher fidelity CR neutron source term, fully capturing the directional distribution of neutrons incident on the Earth’s surface as a function of incident energy for specific sites. Investigation of the extent to which the monitor’s response is isolated from environmental influences will be expanded, considering humidity, temperature, moisture fluctuations in surrounding material and snow and ice accumulation around the housing. The model geometry for future analysis will now be derived directly from the CAD models of the engineered design solution, with manufacturer specified material definitions, to ensure that engineering design concessions do not negatively impact the performance of the monitor. Once fabricated, the monitor’s response function will be simulated and validated experimentally.

## Methods

### Environmental radiation source

A number of options exist to model environmental radiation in radiation transport codes, here we consider the suitability of a few options for the purpose of this study. In MCNP for example, an inbuilt cosmic radiation source capability exists, allowing the user to directly transport cosmic particles or alternatively secondary background particles, sampled from a data file shipped with the code. Two approaches could be utilised for neutrons; either directly sampling the background neutrons, or explicitly sampling the cosmic radiation (high energy protons) and transporting this through a column of atmospheric material to determine the secondary neutron emission profile at a given altitude.

Drawbacks exist with both approaches. The background neutron source implementation in MCNP prevents the spatial and directional distribution of neutron emission from being dependent on the neutron energy. As such, any spatial and directional distribution implemented will be the same for all neutron emission energies. In practice, the directional distribution of background neutrons is highly dependent on energy; in general, higher energy neutrons are more likely to be directed towards the Earth’s surface (downwards from the zenith direction) and lower energy neutrons away (upwards). As the detector response depends on factors such as the moderator and producer thickness, and their optimisation, this effect cannot be neglected.

Transporting the cosmic radiation directly would allow the spatial and directional distributions of secondary neutrons to be recorded on appropriately set up neutron flux tallies. Whilst this approach has the advantage of allowing the user to specify the positional and directional bin resolution, it is computationally expensive due to the charged particle transport. Furthermore, a new source would have to be calculated each time parameters such as longitude and latitude are changed. An alternative approach was adopted to allow for greater flexibility during initial tests. However, for a final design evaluation, where all source parameters are likely to be fixed, a high resolution source term derived using this method would be preferential.

Online data resources such as EXPACS^47^ and MAIRE^27^ provide CR neutron data for a given location and date as a function of energy, resolved into angular bins defined relative to the zenith direction. Data from either of these tools can be generated and easily exported into a .csv file, allowing multiple source terms to be derived from them. Whilst this method offers flexibility with regards to the input data source, EXPACS and MAIRE are both limited by the directional distribution resolution in the data they provide. However, they provide an adequate approximation for testing purposes and relative comparisons between models.

This study used the MAIRE database to generate an environmental source term using the Culham site in Oxfordshire, UK and an arbitrary date for the relative comparisons presented. The .csv file produced by the MAIRE database was converted to an equivalent source definition (sdef) term for use in MCNP using a bespoke Python utility. This sdef term was used for all the analysis presented.

**Figure 7:**
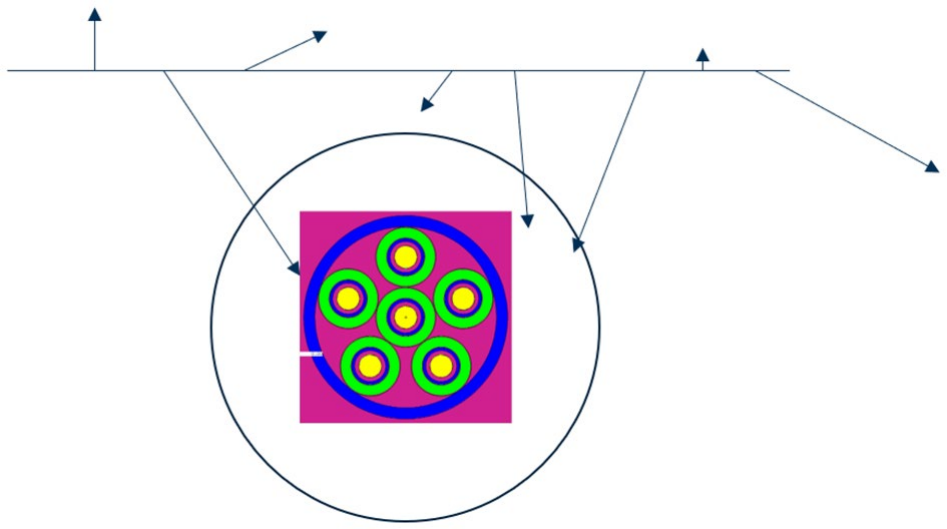
Illustration of the environmental radiation source normalisation methodology. The arrows indicate the direction of neutrons, which are recorded in the sphere in a track length estimator tally to compute a normalisation factor. The source was sampled on a 10 m $\times$ 10 m plane surface, positioned 5 m above the origin of the coordinate system. All models were oriented such that the centre of the monitor was at the origin. Using a void geometry with a sphere cell of radius 2 m centred on the origin, the neutron source definition is sampled and the cell volume averaged neutron flux estimate over the sphere is recorded.

Using a similar methodology adopted in the literature^48^, a separate MCNP calculation was conducted to determine a neutron source definition normalisation factor which would induce a ground level neutron flux of 100 n m^-2^^49^. The source was sampled on a 10 m $\times$ 10 m plane surface, positioned 5 m above the origin of the coordinate system. All models were oriented such that the centre of the monitor was at the origin. Using a void geometry with a sphere cell of radius 2 m centred on the origin, the neutron source definition is sampled and the cell volume averaged neutron flux estimate (f4 tally) over the sphere is recorded; this scores a track length estimate as each neutron passes through the cell. The default units on an f4 tally are n cm^-2^/source particle, therefore the result must be scaled up by a factor of 1000 to convert to n m^-2^ /source particle before deriving a scale factor of 100 n m^-2^ divided by this result. This value is subsequently used as the weight argument on the neutron source definition, therefore normalising any output data generated from the MCNP calculation. Figure 7 provides an example visual illustration of the normalisation calculation, where a dummy PTI-204 BCS tube has been added to illustrate the origin location.

### Development of the BCS model

BCS detectors were evaluated as a possible alternative to BF_3_ and ^3^He gas-filled proportional counters for ground level neutron monitoring, the advantages being to avoid the use of hazardous BF_3_ and expensive ^3^He. One of our related studies evaluated two BCS detector configurations and experimentally benchmarked them against COTS ^3^He detector solutions to validate accompanying MCNP simulations^41^. This study details the development and validation of the BCS model used throughout this study.

### Parameterised model development

Two main parameterisation studies were conducted: a parameterised representation of a simplified 6-NM-64 model, referred to as the cavity model; and a slab model parameterisation. A UKAEA developed Python package, mcnpfilewriter, was used to generate MCNP input files for a defined set of parameters. The package was first modified to accommodate the entry of pre-determined material data and use of custom source routines, i.e., the CR neutron source term described.

Our predominant comparisons are between our new ^3^He-based slab monitor design and the NM-64 BF_3_ cavity design. For the NM-64 BF_3_ case, a ^10^B(n,$\alpha$)^7^Li reaction rate tally was added to the BF_3_ gas volume of each counter. Similarly, for ^3^He gas-filled counters, a ^3^He(n,p)^3^H reaction rate tally was added to the ^3^He gas volume. When considering these gas counters, the reaction products both take place in the gas. This results in spectra for both BF_3_ and ^3^He gas-filled counters that exhibit a clear valley between gamma noise and reaction event. In practice, proportional counters operate in that valley and so discard very few genuine events. Therefore we assumed that the tally result will be equivalent to the count rate observed by each counter, i.e., a 1:1 ratio between products and total counts. In contrast, for the BCS, the reactions take place in the boron carbide deposit on the inner wall of the straw and so there is a possibility the reactions are released in the deposit and never get out into the counter gas. This process means that whatever happens part of the energy is usually lost in the deposit before it gets out. This results in a continuous spectrum meaning a threshold must be applied to discriminate gamma and microphonic noise and necessarily reject some of the events. Therefore, for the BCS detectors, only $\sim$(0.69±0.02) of ^10^B(n,$\alpha$) reactions are counted based on measured/calculated results in a calibration field^41^, this is referred to as the electronic efficiency factor (EEF).

A Python script was set up to provide a user interface that allowed for the parameters to be generated in a random or systematic fashion between user specified minimum and maximum bounds for each parameter. MCNP output data is subsequently read in and stored in memory for each simulation. The Python script then processes the MCNP output data and subsequently writes it to a .csv data file, specifying the count rate calculated in each cavity.

#### Cavity model

A parameterised model was developed to explore key features which affect the sensitivity of the NM-64 design to the neutron source. The model was derived from the NM-64 design with the Pb wings removed to simplify the parameterisation. The inner cavity was then populated with various neutron detectors, including BF_3_, ^3^He and BCS counters. From the cavity outwards, the polyethylene moderator is surrounded by an air gap, which separates it from the Pb producer. An outer air gap was included to separate the outer polyethylene reflector from the Pb producer. The parameters used to confine the model as illustrated in Fig. 1b. For illustrative purposes, the reflector layer has been drawn around a single cavity; 6-counter arrays were produced for the simulations presented.

**Figure 8:**
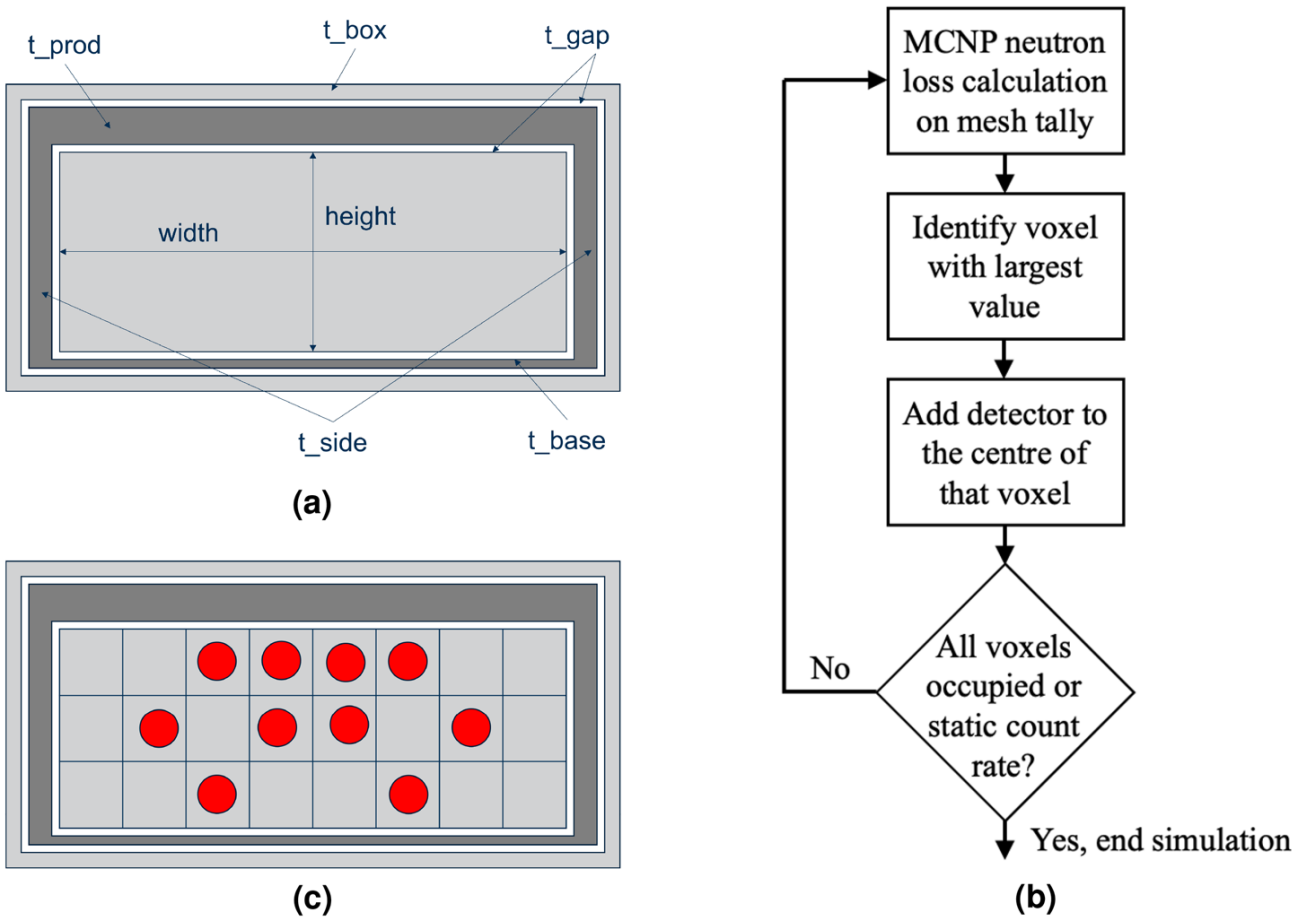
Parameterised slab model and detector location optimisation. (**a**) Schematic of the slab model with key parameters indicated, comprised of the polyethylene outer reflector and inner moderator (light grey regions), and the Pb producer (dark grey). (**b**) Flow diagram of the workflow implemented for detector location optimisation within the slab model. (**c**) Illustrative example of the detector location optimisation workflow in practice.

#### Slab model

To increase the packing density of neutron detectors and reduce neutron depletion in detector locations, a slab concept model was developed to explore the performance of an alternative geometric configuration. It was hypothesised that the concept would have several advantages over the cavity model, including improved performance, simplicity of manufacture and cost benefits afforded by improved utilisation of materials.

The slab consists of a cuboidal polythene moderator, with cylindrical bores to accommodate 1^″^ diameter neutron detectors. The moderator block is surrounded by the Pb producer layer. Beyond this, a second polyethylene casing acts as a reflector to reflect and moderate the evaporation neutrons that are produced in the Pb producer, and to reflect and absorb the low energy neutrons that are produced by high energy nucleons in interactions with the materials surrounding the monitor. The model includes air gaps between the material layers and around the neutron detectors. Figure 8a shows a cross section of the model, illustrating the parameters used in the model (detector bores are excluded).

Two separate schemes of optimisation were performed; one to optimise the slab parameters, and following this, an optimisation of the detector locations within the moderator of the slab model.

## Data Availability

The datasets generated, used and analysed during the current study are available at 10.17635/lancaster/researchdata/639.
